# Mid-term safety and efficacy in small intracranial aneurysm coiling: results from TARGET^®^ nano prospective independent core lab adjudicated multicenter registry

**DOI:** 10.3389/fneur.2024.1325527

**Published:** 2024-05-13

**Authors:** Yazan Ashouri, Alexandra R. Paul, Ajit Puri, Nicholas Liaw, Aniel Majjhoo, Asif Taqi, Ansaar Rai, Aamir Badruddin, Amer Alshekhlee, Bharath Naravetla, Mahmoud Rayes, Matthew Lawson, Batool Al Masaid, Claire Langerford, Qaisar Shah, Karen Beaty, Eugene Lin, Tanner Gray-Duvall, Jasmine Olvany, Hannah Slight, Varun Chaubal, Saif Bushnaq, Benedict Tan, Mohammad Al Majali, Lucas Elijovich, Peter Sunenshine, Osama O. Zaidat

**Affiliations:** ^1^Neuroscience Institute, Bon Secours Mercy Health St. Vincent Hospital, Toledo, OH, United States; ^2^Department of Neurosurgery, Albany Medical Center, Albany, NY, United States; ^3^Department of Radiology, University of Massachusetts, Worcester, MA, United States; ^4^Vascular Neurology Las Vegas, Las Vegas, NV, United States; ^5^McLaren Health, Flint, MI, United States; ^6^McLaren Health, Macomb, MI, United States; ^7^Desert Regional Medical Center, Palm Spring, CA, United States; ^8^Department of Radiology, Neurology, and Neurosurgery, West Virginia University, Morgantown, WV, United States; ^9^Neuroscience Department, Presence St. Joseph Medical Center, Joliet, IL, United States; ^10^Community Healthcare System, Munster, IN, United States; ^11^Greenville Health Systems, Greenville, SC, United States; ^12^Tallahassee Neurological Clinic, Tallahassee, FL, United States; ^13^MedX Cro LLC, Kent, DE, United States; ^14^Abington Memorial Hospital, Abington, PA, United States; ^15^St. George’s University SOM, True Blue, Grenada; ^16^Department of Neurology, Semmes Murphey, Memphis, TN, United States; ^17^Banner University Medical Center, Phoenix, AZ, United States

**Keywords:** intracranial aneurysm, coiling, TARGET, nano coils, safety, efficacy

## Abstract

**Background:**

The primary objective is to evaluate the safety and effectiveness of Stryker second generation Target^®^ Nano Coils in the treatment of ruptured and unruptured small (<7 mm) intracranial aneurysms.

**Methods:**

The TARGET Registry is a prospective, two-arm study with independent medical event monitoring and core-lab adjudication. This paper describes the second arm of the TARGET registry. Patients with *de novo* intracranial aneurysms were embolized with 2nd generation TARGET Nano coils in 12 US centers. The primary efficacy outcome was adequate aneurysm occlusion (RR occlusion grade I-II) on follow-up. Primary safety outcome was treatment-related morbidity and mortality. Secondary outcomes included aneurysm packing density immediately post-procedure, immediate adequate occlusion, aneurysm re-access rate, retreatment rate and clinical outcomes using modified ranking scale. A secondary analysis investigated the influence of using Nano-predominant coils (≥2/3 of total coil-length) vs. non-Nano-predominant coils (<2/3 of total length).

**Results:**

150 patients with 155 aneurysms met the inclusion and exclusion criteria. (31%) patients with ruptured and (69%) with unruptured aneurysms were treated using TARGET coils. Median age was 58.8 (SD 12.7), 74.7% were females, and 80% were Caucasians. Mean follow-up was 5.23 (SD 2.27) months. Peri-procedural mortality was seen in 2.0% of patients. Good outcome at discharge (mRS 0–2) was seen in 81.3% of the cohort. The median packing density (SD) was 29.4% (14.9). Mid-term complete/near complete occlusion rate was seen in 96% of aneurysms and complete obliteration was seen in 75.2% of aneurysms. Patients treated predominantly with Nano coils had higher PD (32.6% vs. 26.1%, *p* < 0.001). There was no significant difference in clinical and angiographic outcomes. The mid-term mRS0-2 was achieved in 106/109 (97.2%) patients. All-cause mortality was 5/115 (4.3%).

**Conclusion:**

In the multicenter TARGET Registry, 75.8% of aneurysms achieved mid-term complete occlusion, and 96% achieved complete/near complete occlusion with excellent independent functional outcome.

## Introduction

Intracranial aneurysms (IA) have a prevalence of 2.0–3.2% in the general population with a 1.9% risk of rupture annually (1–3). These events carry significant morbidity and mortality. The ATENA study found that aneurysms <10 mm represent 89% and 76.7% of unruptured anterior and posterior circulation IA, respectively (4).

Since the publication of the ISAT and BRAT trials, endovascular coiling has been evolving with the development of softer and smaller coils leading to safer and more efficient treatments of intracranial aneurysms (5, 6). Three-dimensional coils have been demonstrated to lower rates of recanalization and provide better aneurysmal occlusion (7, 8). The Target 3D Nano coils (Stryker Neurovascular, Fremont, CA, United States) represent an advancement in soft coil mechanisms. The ULTRA registry reported safe and effective outcomes in very small IA (<5 mm) treated with Nano coils (9). However, evidence of the safety and efficacy of Nano coils in small aneurysms <7 mm is not well established.

The TARGET Intracranial Aneurysm Coiling (TARGET) Registry was a prospective, investigator-initiated, non-randomized, multicenter study with an independent clinical event and core-lab adjudication, which aimed to collect real-world data on Target^®^ 360° and Target^®^ helical coils for the embolization of ruptured and unruptured intracranial aneurysms. The results of the first TARGET registry results were previously published by Zaidat et al. indicating the safety and efficacy of the Target 360 and helical coils in treating small aneurysms (10). Here, we present the second arm of the peri-procedural, mid-term safety and occlusion efficacy results from the TARGET Nano Registry which enrolled a new population of patients with the aim of establishing the safety and efficacy in small intracranial aneurysms using very soft Target Nano Coils.

## Materials and methods

### Study design

The prospective, multicenter TARGET Nano Registry included ruptured or unruptured small (≤7 mm) saccular intracranial aneurysms, which were treated with at least 25% Nano coils (ClinicalTrials.gov Identifier: NCT01748903). The focus of this arm of the prospective registry is the safety in using super ultrasoft coils in small cerebral aneurysms.

A total of 12 clinical sites within the United States with active IRB approval to participate in the TARGET Registry with 11 centers enrolling at least 1 patient, the median [IQR] aneurysms per clinical site was 12 [6–24]. Mercy St. Vincent Medical Center, Toledo, Ohio served as the coordinating center for TARGET Nano Registry.

### Study population

Patients were eligible for enrollment in the TARGET Nano Registry if the following criteria were met: (1) Patient is 18 years or older. (2) Patient has a documented, previously untreated, saccular intracranial aneurysm, unruptured or ruptured, suitable for embolization with coils. (3) Index aneurysm is ≤7 mm. (4) Patient has a Hunt and Hess Score of 3 or less. (5) Patient has a premorbid mRS of 3 or less. (6) Patient or patient’s legally authorized representative has provided written informed consent. (7) Patient is willing to and can comply with study follow-up requirements. Patients were excluded from enrollment if they met any of the following: (1) Less than 18 years of age, (2) non-saccular aneurysms, (3) Patients with intracranial aneurysms (other than the index aneurysm) that will require treatment during the study period (enrollment through follow-up), (4) Patients in whom the index aneurysm was treated with a total coil length comprised of <25% Stryker Target® 2nd generation Nano Coils., and (5) Patients in which the index aneurysm cannot be coiled in one procedure (i.e., staged procedure).

### Study arms (predominantly nano vs. nano as finishing coils)

Standard local institutional trans-arterial coiling techniques and anesthesia were used at each site per the participating interventionalist preference. Given that the secondary intention of this study was to assess the results of aneurysms based on coil softness, patients treated predominantly with Nano coils (≥2/3 Nano coils) were compared with those treated with <2/3 Nano coils. Investigators were encouraged to use the same coil type for framing, filling, and finishing, if possible.

The use of adjunctive devices was permitted. Mid-term follow-up occurred at 3–9 months (per each site’s standard of care) and included imaging of the index aneurysm (DSA or MRA) and assessment of clinical outcome using the modified Rankin Scale (mRS). All source data from case reports were entered into a secure database (REDCAP) and managed/monitored by the central coordinating center.

### Clinical and imaging adjudication

An independent medical monitor reviewed all adverse events (AEs) and serious AEs (SAEs) for the study and adjudicated their relatedness to the underlying diseases, procedure, or study device.

For determination of aneurysm occlusion outcome, de-identified vascular angiographic images (DSA and MRA) were sent to the core imaging lab for adjudication of the modified Raymond aneurysm occlusion grade immediately post-procedure and on mid-term imaging follow-up (11, 12). The progression to complete occlusion, or regression to aneurysm recurrence and recanalization was also assessed by the core imaging lab.

### Study outcomes

The primary efficacy outcome was adequate aneurysm occlusion (RR occlusion grade I-II) on follow-up. Secondary efficacy outcomes were aneurysm packing density immediately post-procedure, microcatheter kick back rate (requiring aneurysm re-access), time of fluoroscopic exposure, overall procedure time, aneurysm recurrence at follow-up and aneurysm re-treatment rate. Primary safety outcome was treatment-related morbidity and mortality. Other study outcomes were clinical outcome (mRS) at follow-up, all-cause mortality and peri/post-procedural adverse events related to device and/or procedure. Symptomatic TE events were defined as persistent (>24 h) neurological deficit secondary to documented thromboembolic event with worsening of NIHSS>4 points.

A secondary analysis was performed comparing technical and clinical endpoints between aneurysms in patients treated with Nano-predominant coils (≥2/3 total coil length) vs. those treated with non-Nano predominant coils (<2/3 total coil length), this analysis was not predefined in the study protocol.

### Statistical methods

Categorical data is presented as a percentage for all patients (*n* = frequency count) and as a percentage by treatment group (rounded to the nearest whole number). Continuous data is expressed as mean with standard deviation (SD) or median with interquartile range [IQR] if non-normally distributed. Categorical factors were compared between treatment groups with Chi-square tests or Fisher’s Exact tests when cell sizes were small. Continuous data were compared. All statistical tests were 2-sided and a *p*-value <0.05 was considered significant. All statistical analyses were performed using IBM SPSS version 28.0 For Windows (Armonk, NY: IBM Corp).

This study was not designed to detect differences of a certain magnitude between groups with *a priori* power calculation. Therefore, the lack of a significant p-value (i.e., “no significant difference”) may be due to the small sample size.

## Results

A total of 150 patients with 155 ruptured or unruptured aneurysms were prospectively enrolled and included in the analysis. Patients’ characteristics and demographics are outlined in Table 1. The mean age (SD) of the cohort was 58.8 (12.7) years old. The majority of patients were females (74.7%) and (80%) were Caucasians. The median [IQR] aneurysm size was 5.0 [3.96–5.87] mm with most aneurysms presenting in the anterior circulation (85.7%; Table 2).

**Table 1:** Overall cohort characteristics and demographics.

Variable	Total *n* = 150	Nano <66.6% N = 89 (59.3%)	Nano >66.67 *N* = 61 (40.7%)	*p* value
Age Mean (SD)	58.8 (12.7)	58 (13.3)	59.9 (11.8)	0.379
Gender, Female	112 (74.7%)	70 (78.7%)	42 (68.9%)	0.187
Caucasian	120 (80%)	67 (75.3%)	53 (86.9%)	0.135
AA	19 (12.7%)	15 (18%)	3 (4.9%)	
Hispanic	7 (4.3%)	4 (4.5%)	3 (4.9%)	
Headache/migraine	78 (53.1%)	43 (48.9%)	35 (59.3%)	0.213
History of Hemorrhagic	5 (3.4%)	4 (4.5$)	1 (1.7%)	0.649
History of Ischemic	23 (15.4%)	13 (14.6%)	10 (16.7%)	0.733
TIA	8 (5.4%)	4 (4.5%)	4 (6.7%)	0.715
Family history of ICA	37 (26.1%)	24 (28.6%)	13 (22.4%)	0.411
Diabetes Mellitus	29 (19.5%)	17 (19.3%)	12 (19.7%)	0.957
Hypertension	102 (68%)	64 (71.9%)	38 (62.3%)	0.215
SMOKING	72 (48%)	42 (47.2%)	39 (49.2%)	0.811
Arteriovenous malformation	1 (0.7%)	1 (1.1%)	0.0	1.000
Hyperlipidemia	62 (41.3%)	35 (39.3%)	27 (44.3%)	0.546
Previously treated	17 (11.3%)	9 (10.1%)	8 (13.1%)	0.569
Premorbid mRS				
0	102 (82.3%)	64 (86.5%)	38 (76%)	0.022
1	15 (12.1%)	4 (5.4%)	11 (22%)	
2	4 (3.2%)	3 (4.1%)	1 (2.0%)	
3	3 (2.4%)	3 (4.1%)	0	

SD, Standard deviation; AA, African American; TIA, Transient ischemic attack; mRS, Modified rankin scale.

**Table 2:** Pre-treatment aneurysm evaluation: overall cohort.

Variable	Total *n* = 155	Nano <66.6% *N* = 92 (59.3%)	Nano >66.67 *N* = 63 (40.7%)	*p* value
Aneurysm size mm, median (IQR)	5.0 (3.96–5.87)	5.01 (4.0–6.13)	4.81 (3.84–5.48)	0.03
Baseline mRS				
0	106 (70.7%)	68 (76.4%)	38 (62.3%)	0.06
1	22 (14.7%)	7 (7.9%)	15 (24.6%)	
2	9 (6%)	7 (7.9%)	2 (3.3%)	
3	9 (6%)	5 (5.6%)	4 (6.6%)	
4	1 (0.7%)	1 (1.1%)	0	
5	3 (2.0%)	1 (1.1%)	2 (3.3%)	
Aneurysm Volume Median (IQR)	35.1 (18.31–59.91)	38.5 (24.06–76.05)	32.07 (16.83–51.16)	0.02
Neck Diameter Mean (SD)	2.94 (1.48)	2.74 (1.03)	3.25 (1.95)	0.0
Multiple Aneurysm	5 (3.2%)	1 (1.1%)	45 (6.3%)	0.04
Side: Right	79 (51%)	46 (50%)	33 (52.4%)	0.849
Left	57 (37.4%)	36 (39.1%)	22 (34.91%)	
Midline	18 (12.1%)	10 (11.4%)	8 (13.1%)	
Location				
ICA	57 (36.8%)	37 (40.2%)	20 (31.7%)	0.296
Acom	48 (31%)	26 (28.3%)	22 (34.9%)	
MCA	26 (16.8%)	14 (15.2%)	12 (19%)	
Basilar	18 (11.6%)	13 (14.1%)	5 (7.9%)	
Vertebral	3 (1.9%)	2 (2.2%)	1 (1.6%)	
Extradural	2 (1.3%)	0 (0%)	2 (3.2%)	
Bifurcation	88 (56.8%)	51 (55.4%)	37 (58.7%)	0.740
Anterior	132 (85.7%)	78 (84.8%)	54 (87.1%)	0.687
Irregular shape	80 (51.9%)	41 (44.6%)	39 (62.9%)	0.025
Ruptured	48 (31%)	24 (26.1%)	24 (38.1%)	0.112
HH Scale				
I	7 (14.6%)	3 (12.0%)	4 (17.4%)	0.028
II	26 (54.2%)	18 (72%)	8 (34.8%)	
III	15 (31.3%)	4 (16.0%)	11 (47.8%)	
Technical Feature Stent-assisted	72 (46.5%)	44 (47.8%)	28 (44.4%)	0.678
Stent TYPE:				
Atlas	32 (44.4%)	15 (34.1%)	17 (60.7%)	0.083
EZ	24 (33.3%)	17 (38.6%)	7 (25%)	
Others	16 (22.2%)	12 (27.3%)	4 (14.3%)	
Balloon assisted	18 (11.6%)	9 (9.8%)	9 (14.3%)	0.390
Hyperform	2 (11.1%)	1 (11.1%)	1 (11.1%)	0.650
Hyperglide	1 (5.6%)	0 (0%)	1 (11.1%)	
SCEPTER	7 (38.9%)	3 (33.3%)	4 (44.4%)	
Transform	8 (44.4%)	5 (55.6%)	3 (33.3%)	
Total procedural time Median (IQR) minutes	90.5 (68.0–120.5)	83.5 (68–118)	95 (29–127.50)	0.338
Total fluoroscopic time Median (IQR) minutes	26 (17–40)	25 (16–38.0)	29 (21.5–42.50)	0.057

mRS, Modified rankin scale; IQR, Interquartile range; ICA, internal carotid artery; ACom, Anterior communicating artery; MCA, Middle cerebral artery; HH, Hunt & Hess.

The most common aneurysm location was the ICA (36.8%) followed by the ACom (31%). A total of 48 (31%) of the treated aneurysms were ruptured. Of the total cohort, 72 patients (46.5%) were treated with stent-assisted coiling and 18 patients (11.6%) were treated with balloon-assisted coiling. The median [IQR] procedural time was 90.5 [68–120.5] minutes, and the median [IQR] fluoroscopic time was 26 [17–40] minutes.

The mean (SD) packing density was 29.4% (14.5; Table 3). Immediate adequate occlusion (Modified Raymond Roy (RR) occlusion grade I-II) was seen in 95.5% of cases, where the remaining 4.5% had incomplete occlusions. Immediate complete occlusion was achieved in 62.6% of the cohort.

**Table 3:** Immediate and mid-term post-treatment outcome.

Variable	Total *n* = 150 Aneurysms: 155	Nano <66.6% *N* = 89 (59.3%) Aneurysm: 92	Nano >66.67 *N* = 61 (40.7%) Aneurysms: 63	*p* value
Packing density mean (SD)	29.4 (14.5)	26.05 (10.78)	34.19 (17.58)	<0.001
Immediate RROC				
I	97 (62.6%)	58 (63.0%)	39 (61.9%)	0.903
II	51 (32.9%)	30 (32.6%)	21 (33.3%)	
I-II	148 (95.5%)	88 (95.7%)	60 (95.2%)	
Discharge mRS				
0–2	122 (81.3%)	73 (82.2%)	49 (80.3%)	0.794
0–1	112 (74.4%)	68 (76.4%)	44 (72.1%)	0.554
Dispo				
Home	105 (70%)	67 (75.3%)	38 (62.3%)	0.259
Rehab	8 (5.3%)	4 (4.5%)	4 (6.6%)	
ECF	4 (2.7%)	3 (3.4%)	1 (1.6%)	
Hospice	1 (0.7%)	1 (1.1%)	0 (0%)	
Nursing home	2 (1.3%)	0 (0.0%)	2 (3.3%)	
Other	27 (18%)	12 (13.5%)	15 (24.6%)	
Periprocedural mortality	3 (2.0%)	0 (0%)	3 (4.9%)	0.068
Device/procedural-associated Adverse event	2 (1.3%)	2 (2.2%)	0 (0.0%)	0.120
Microcatheter kick-back	22 (14.7%)	11 (12.4%)	11 (18%)	0.349
Symptomatic TE event	1 (0.7%)	1 (1.1%)	0 (0%)	0.140
Asymptomatic TE event	4 (2.7%)	1 (1.1%)	3 (4.9%)	
Stroke	3 (2.0%)	1 (1.1%)	2 (3.3%)	
Asymptomatic intraoperative perforation	1 (0.7%)	0 (0%)	1 (1.6%)	
SAH > 24H from procedure	2 (1.3%)	1 (1.1%)	1 (1.6%)	1.00
All-cause Mortality *n* = 115	5 (4.3%)	1 (1.5%)	4 (8.3%)	0.158
Mean follow-up duration, months	5.23 (2.27)	5.47 (2.24)	5 (1.96)	0.284
Retreatment Rate (*n* = 114)	1 (0.9%)	1 (1.4%)	0 (0%)	1.000
Will require retreatment? (*n* = 112)	5 (4.5%)	4 (5.9%)	1 (2.3%)	0.611
Follow-up imaging modality				
DSA	90 (75%)	53 (71.6%)	37 (80.4%)	0.378
MRA	30 (25%)	21 (28.4%)	9 (19.6%)	
Mid-term RROC (*n* = 125)				
I	94 (75.2%)	59 (77.6%)	35 (71.4%)	0.825
II	26 (20.8%)	14 (18.4%)	12 (24.5%)	
I-II	120 (96%)	73 (96.1%)	47 (95.9%)	1.000
Mid-term RROC I-II per treatment method				
Balloon-assisted coiling (*n* = 14)	13 (92.9%)	13 (92.9%)	13 (92.9%)	1.00
Primary coiling (*n* = 53)	51 (96.2%)	51 (96.2%)	51 (96.2%)	
Stent-assisted coiling (*n* = 58)	56 (96.6%)	56 (96.6%)	56 (96.6%)	
Occlusion Prog (*n* = 122)				
Better	49 (40.2%)	27 (36.5%)	22 (45.8%)	0.488
Stable	62 (50.8%)	39 (52.7%)	23 (47.9%)	
Worse	11 (9%)	8 (10.8%)	3 (6.3%)	
Mid-term MRS (*n* = 109)				
mRS0-2	106 (97.2%)	63 (96.9%)	43 (97.7%)	1.000
mRS0-1	97 (89%)	55 (84.6%)	42 (95.5%)	0.119
Adverse event since discharge	16 (14%)	8 (11.4%)	8 (18.2%)	0.312

SD, Standard deviation; RROC, Raymond-Roy occlusion Criteria; mRS, Modified rankin scale; SAH, Subarachnoid hemorrhage; TE, Thromboembolic.

Microcatheter kickback (requiring re-accessing of the aneurysm) occurred in 22 patients (14.7%). Symptomatic thromboembolic (TE) events occurred in 1 patient (0.7%) and asymptomatic TE events occurred in 4 patients (2.7%), while 3 patients had a permanent deficit (2%). Asymptomatic intra-operative perforation (IOP) occurred in 1(0.7%) patient within the Nano-predominant coiling.

**Figure 1:**
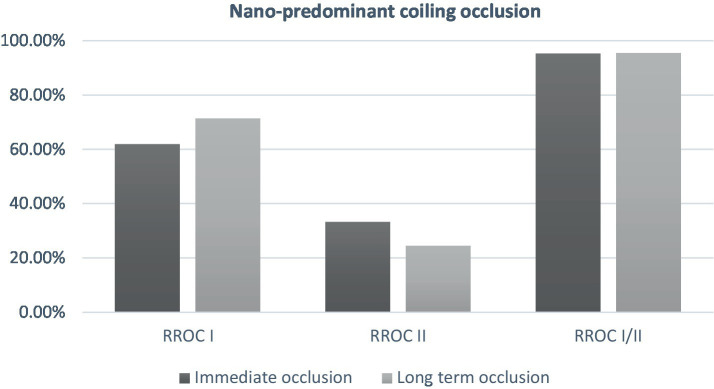
Immediate and mid-term occlusion in patient treated with nano-predominant coiling (≥2/3 of total coil length).

Two (1.3%) patients had device-related adverse events (DAE). In the first patient, fter deployment of the final finishing coil, while trying to maneuver back into aneurysm coil prematurely detached in the catheter. This necessitate placing a second Atlas stent, while the other patient had a vasospasm in the ACA requiring verapamil. Both patients were within the non-Nano predominant coiling. A total of 3 (2.0%) patients expired in the hospital (2 ruptured aneurysms and 1 unruptured aneurysms). Patient who expired with an underlying unruptured wide-neck Acomm irregular aneurysm was treated using SAC who developed procedure-related SAH, followed by family decision to withdraw care and patient expired 3 days post-procedure/event. In terms of ruptured aneurysm patients, a 66-year-old female with a ruptured pcomm aneurysm and HH score of 3 had an underlying aortic stenosis and developed acute pulmonary edema and respiratory failure on the 9^th^ day of hospitalization. Another 88-year-old female with one ruptured pcomm aneurysm and HH score of 1 with an underlying atrial fibrillation, developed cardiac arrest unrelated to procedure on the 8^th^ day of hospitalization. Good functional outcome (mRS 0–2) at discharge was seen in 81.3% of patients and the majority of patients (83.2%) with unruptured aneurysms vs. almost one third of ruptured aneurysms (33.3%) were discharged home (Figure 1).

**Figure 2:**
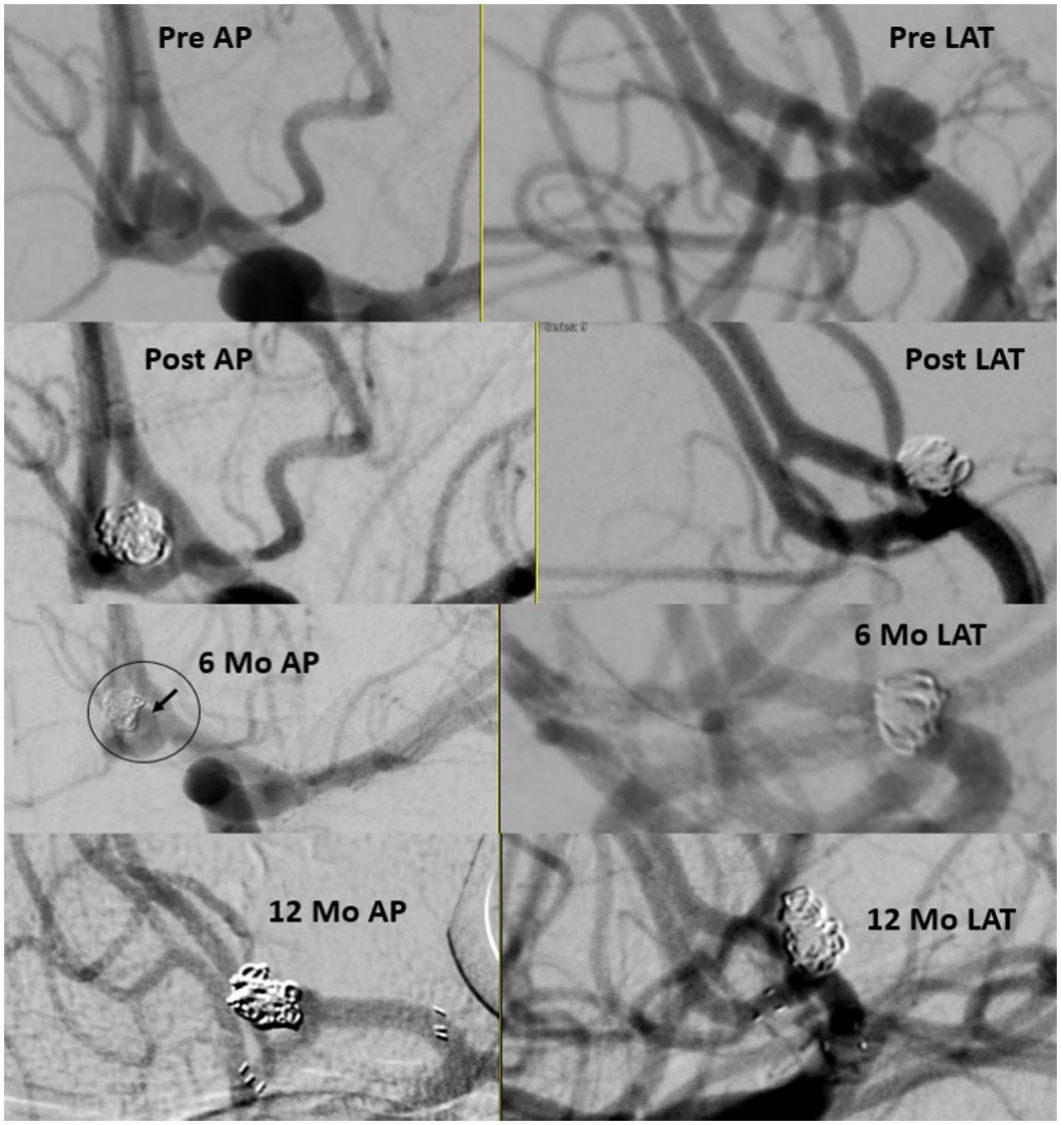
Ruptured small A-comm 3.79 mm in height and 2.78 in width with 2.3 mm neck. Immediate results with neck on 6 months follow up treated with stent assisted coiling with stable occlusion 1 year later.

**Figure 3:**
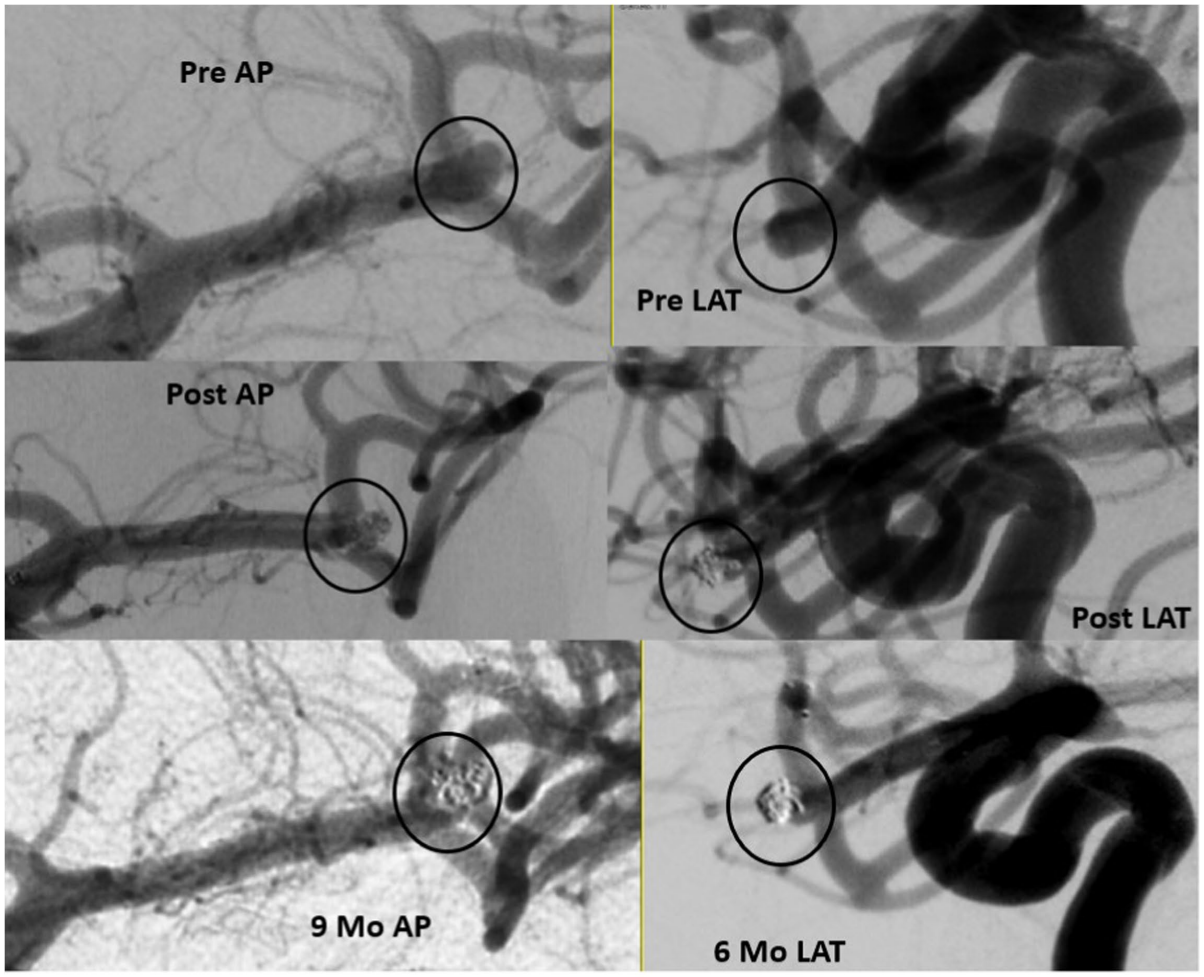
Ruptured small MCA 3.63 mm in height and 2.54in width with 2.1 mm neck (upper panel). Immediate results with filling in the interstices (middle panel) and 9 months follow up (lower panel).

Of the total cohort, 109 patients (72.7%) had clinical follow-up data and 125 aneurysms (80.6%) had angiographic follow-up data (Table 3). Most patients underwent DSA for follow-up imaging (n = 90, 75%). The mean (SD) follow-up duration was 5.29 (2.28) months. Midterm adequate occlusion (RR I-II) was achieved in 96% of patients, while complete aneurysm obliteration (RR I) was achieved in 75.2% (Figures 2, 3). The overall retreatment rate through follow-up was 0.9 and 4.5% of patients will require future retreatment. The rate of good functional outcome was 97.2% and the all-cause mortality rate was 4.3%.

### Nano-predominant coiling vs. other coiling

A secondary analysis comparing a Nano-predominant cohort (≥2/3 Nano coil, *n* = 61 patients [63 aneurysms]) to a non-Nano predominant cohort (<2/3 Nano coil *n* = 89 patients [82 aneurysms]) was performed in Table 1. There were no significant differences between the two groups, except for a trend in the distribution of premorbid mRS score.

The mean maximum aneurysm size was significantly smaller in the Nano-predominant group (median [IQR] = 4.81 (3.94–5.48) vs. 5.01 (4.0–6.13), *p* = 0.03) when compared to non-Nano-predominant group in Table 2. Moreover, the Nano-predominant group had significantly smaller aneurysm volume (median [IQR] = 32.07[16.83–51.16] vs. 38.5[24.06–76.05], *p* = 0.02). The mean aneurysm neck diameter was similar between the two groups. Furthermore, there were no significant differences in the location of the aneurysm or in relation to a branching point (bifurcation vs. sidewall). However, patients treated within the Nano-predominant group were more likely to have an irregular aneurysm shape (62.9% vs. 44.6%, *p* = 0.025; Table 2). Although aneurysms from both groups were similar in terms of rupture status, patients within Nano-predominant group were more likely to have grade III HH score (38.1% vs. 26.1%, *p* = 0.028) when compared to the non-Nano-predominant group. The median procedural time was similar between the two groups, with a trend to longer median fluoroscopic time in the Nano-predominant group when compared to the non-Nano-predominant group (29 vs. 25 min, *p* = 0.057).

The mean (SD) packing density was significantly higher in the Nano-predominant group 34.19 (17.58) vs. 26.05 (10.78) in the non-Nano predominant group (*p* < 0.001; Table 3). No difference was observed in the immediate post-treatment occlusion rates or in the rates of good clinical outcomes between the two subgroups.

At follow-up, no significant difference was observed in the rates of complete to near complete occlusion (Table 3). The complete occlusion rate was similar between the non-Nano predominant group and the Nano predominant group at 77.6 and 71.4%, respectively (*p* = 0.825; Figure 1). Subgroup analysis of patients who underwent primary coiling or BAC, yielded consistent findings. There was no statistical difference was seen in the re-treatment rate between the two groups (1.4 and 0%, *p* = 1.0).

The rate of good clinical outcome (mRS 0–2) at follow-up was 96.9% in the non-Nano-predominant group vs. 97.7% in the Nano-predominant group, *p* = 1.00 (Table 3).

Of the total cohort 10 patients harbored aneurysms with maximum diameter between 7 and 8.4 mm. Therefore, a sensitivity analysis was conducted after excluding patients who had aneurysms ≥7 mm. There were no differences observed in terms of complete and adequate occlusion as well as packing density. Moreover, the rate of good functional outcome remained consistent (Analysis not shown).

## Discussion

Coil embolization remains the mainstay choice for the endovascular treatment of small IA. In a large systematic review, authors reported overall better immediate adequate occlusion for bare platinum coils vs. modified coils, and similar occlusion and retreatment rates during follow-up (13). Therefore, multiple advancements and efforts have been made to improve the softness, structure, and safety of bare platinum coils. As previously described, the Target coils provide a softer distal push wire and a more supportive proximal wire, with smaller coil diameters compared to previous generations.

The current study provides clinical evidence from the TARGET registry of the safety and efficacy of Target coils in small intracranial aneurysms treated with helical and 360 coil systems. In keeping with the findings reported by Zaidat et al. in this first TARGET registry arm, small aneurysms were safely and effectively treated with Target 3D or Helical Coils (10). A high percentage of patients (96%) achieved adequate occlusion on follow-up and almost all patients (97.2%) had good long term functional outcome (mRS 0–2). The rate of angiographic outcome remained consistent even after excluding stent-assisted coiling. The association of SAC with less recurrence in ruptured and unruptured aneurysms has been previously described (14, 15).

In terms of outcomes, rates of device-associated adverse events (1.3%), retreatment (0.9%) and planned future retreatment (4.5%) were relatively low. An intraoperative rupture occurred in 1 patient and 3.4% of patients had TE complications while 3 patients developed permanent deficit. Periprocedural mortality occurred in 3 patients (2.0%). These findings are comparable to previous large studies examining endovascular coiling. The ATENA study reported a rate of 7.3% of TE in patients treated with endovascular coiling with 2.6% rate of intraoperative rupture (4). Although in this series, thromboembolic events occurred in 5.4% and device-related adverse events in 1.3%. Additionally, intraoperative rupture occurred in 1 case (0.7%) and was asymptomatic. The rate of hemorrhagic complication was also similar to that reported in the recent meta-analysis by Algra et al. (0.9%). Although the fatality rate was higher than that reported in the aforementioned study. More recently, the ARETA study reported a rate of TE complications of 10.4% in a large cohort of 1,088 patients and intraoperative rupture in 3.1% (16). The differences in outcomes in this current study may be due to the variation between the registry population vs. the ARETA and ATENA populations, as well as a significantly smaller sample size in our study. Moreover, the rate of good functional outcomes was in line with those of the ISAT trial (5).

Findings from the current study are comparable to other prospective coiling registries using different manufacturer coils (17–19). For instance, Spiotta et al. investigated 172 patients harboring aneurysms ≤4 mm treated with SMART coils (Penumbra, Inc) and reported 97.2% adequate occlusion with 5.6% retreatment rate during a 1-year follow-up. Good functional outcome was observed 86.6% of the time. In the current study, the mean aneurysm size was larger with comparable adequate occlusion (96%) and retreatment rates (0.9%) (17). However, the aforementioned study had a longer mean follow-up duration, compared to the mid-term follow-up in the TARGET registry. Similarly, Fargen et al. investigated 100 patients treated with Axium MicroFX Coils (ev3; Plymouth, Minnesota, United States) and reported an adequate occlusion rate of 90.6%, with a mean follow-up of 5.2 months and 93.3% of patients achieving good functional outcome (mRS 0–2), which is similar to the 97.2% of patients in our series with a good mid-term functional outcome (19). The Trufill DCS Orbit Detachable Coil (Cerenovus, CA) aneurysm registry reported a near complete occlusion rate of 84%, while 5% of aneurysms required retreatment. On follow-up, the rate of good functional outcome was 93.1% (18). In the MAPS trial, the rate of complete and near complete (residual neck) obliteration in patients treated with bare metal coils (BMC) was 39.9 and 27.8%, respectively, for a near complete occlusion rate of 67.7% (20).

### Coil softness and outcomes

In order to further investigate the impact of predominantly Nano coil embolized aneurysms, a secondary analysis was performed to provide a comparison between patients treated with ≥2/3 Nano coil vs. <2/3 Nano coils. The nano coils are designed primarily for softness which may decrease the risk of intraoperative rupture as well as microcatheter kickback. The main disadvantage is related to a propensity toward coil compaction, related to their design for softness. This propensity toward compaction however can be overcome by increased packing (9). With a small sample size, there were no differences in midterm functional outcomes nor in peadequate occlusion between the two groups. However, treatment with ≥2/3 Nano coils did result in a higher packing density. The relationship between packing density and recurrence is currently being debated in the literature, but historically has been thought to be inversely related to aneurysm recurrence (21, 22). The ULTRA registry’s core lab reported a major recurrence rate of 13% between 6 and 9 months, of which 10% required retreatment (9). The authors found that this was lower than the rates for bare platinum coiling and attributed this finding to the increased mean packing density of 34% compared to 24.7%. Kim and colleagues, in a propensity-score matched analysis, investigated outcomes in patients treated with Target Nano coils vs. The Galaxy G3TM MINI micro-coil (GM; Cerenovus, Irvine, CA, United States) (23). Authors reported a safe profile for patients treated with Target Nano coils, with comparable PD to the current registry.

## Limitations

Our study has several limitations including the inherent bias to a registry with non-consecutive patients as well as the lack of a cohort matched control group. In addition, the coil choice was made according to the operator’s preference. Finally, the small sample size and less than one-year mid-term follow-up may have affected some of the negative findings. Future studies will continue to focus on any potential differences in long term recurrence and adequate occlusion in predominantly Nano-coiled aneurysms.

## Conclusion

Overall, we demonstrated that the Target Nano coils can be used in the treatment of small (<7 mm) aneurysms resulting in low complication rates, and mid-term excellent adequate occlusion, low retreatment rates and good functional outcomes.

## Data availability statement

The original contributions presented in the study are included in the article/supplementary material, further inquiries can be directed to the corresponding author.

## Ethics statement

The studies involving humans were approved by IRB Mercy Health St. Vincent West Virginia University Presence St. Joseph Medical Center University of South Carolina SSM DePaul Health Abington Memorial Hospital Michigan State University Gundersen Lutheran Desert Regional Medical Center Texas Stroke Institute University of Massachusetts. The studies were conducted in accordance with the local legislation and institutional requirements. The participants provided their written informed consent to participate in this study.

## Author contributions

YA: Conceptualization, Investigation, Writing – original draft, Data curation, Formal analysis, Methodology, Software, Validation, Visualization, Writing – review & editing. ARP: Writing – original draft, Writing – review & editing. AP: Writing – review & editing. NL: Writing – review & editing. AM: Writing – review & editing. AT: Writing – review & editing. AR: Writing – review & editing. AB: Writing – review & editing. AA: Writing – review & editing. BN: Writing – review & editing. MR: Writing – review & editing. ML: Writing – review & editing. BM: Writing – review & editing. CL: Writing – review & editing. QS: Writing – review & editing. KB: Writing – review & editing. MM: Writing – review & editing. EL: Writing – review & editing. TG-D: Writing – review & editing. JO: Writing – review & editing. HS: Writing – review & editing. VC: Writing – review & editing. BT: Writing – review & editing. LE: Writing – review & editing. PS: Writing – review & editing. OZ: Conceptualization, Investigation, Supervision, Validation, Visualization, Writing – original draft, Writing – review & editing. SB: Writing – review & editing.
